# Netrin-1 Promotes Visceral Adipose Tissue Inflammation in Obesity and Is Associated with Insulin Resistance

**DOI:** 10.3390/nu14204372

**Published:** 2022-10-18

**Authors:** Amaia Mentxaka, Javier Gómez-Ambrosi, Beatriz Ramírez, Amaia Rodríguez, Sara Becerril, Gabriela Neira, Víctor Valentí, Rafael Moncada, Camilo Silva, Xabier Unamuno, Javier A. Cienfuegos, Javier Escalada, Gema Frühbeck, Victoria Catalán

**Affiliations:** 1 Metabolic Research Laboratory, Clínica Universidad de Navarra, 31008 Pamplona, Spain; 2 CIBER Fisiopatología de la Obesidad y Nutrición (CIBEROBN), Instituto de Salud Carlos III, 31008 Pamplona, Spain; 3 Obesity and Adipobiology Group, Instituto de Investigación Sanitaria de Navarra (IdiSNA), 31008 Pamplona, Spain; 4 Department of Surgery, Clínica Universidad de Navarra, 31008 Pamplona, Spain; 5 Department of Anesthesia, Clínica Universidad de Navarra, 31008 Pamplona, Spain; 6 Department of Endocrinology & Nutrition, Clínica Universidad de Navarra, 31008 Pamplona, Spain

**Keywords:** NTN-1, NEO-1, weight loss, Roux-en-Y gastric bypass, caloric restriction, macrophages

## Abstract

Netrin (NTN)-1 exhibits pro- and anti-inflammatory roles in different settings, playing important roles in the obesity-associated low-grade chronic inflammation. We aimed to determine the impact of NTN-1 on obesity and obesity-associated type 2 diabetes, as well as its role in visceral adipose tissue (VAT) inflammation. A total of 91 subjects were enrolled in this case-control study. Circulating levels of NTN-1 and its receptor neogenin (NEO)-1 were determined before and after weight loss achieved by caloric restriction and bariatric surgery. mRNA levels of *NTN1* and *NEO1* were assessed in human VAT, liver, and peripheral blood mononuclear cells. In vitro studies in human visceral adipocytes and human monocytic leukemia cells (THP-1)-derived macrophages were performed to analyze the impact of inflammation-related mediators on the gene expression levels of *NTN1* and its receptor *NEO1* as well as the effect of NTN-1 on inflammation. Increased (*p* < 0.001) circulating concentrations of NTN-1 in obesity decreased (*p* < 0.05) after diet-induced weight loss being also associated with a reduction in glucose (*p* < 0.01) and insulin levels (*p* < 0.05). Gene expression levels of *NTN1* and *NEO1* were upregulated (*p* < 0.05) in the VAT from patients with obesity with the highest expression in the stromovascular fraction cells compared with mature adipocytes (*p* < 0.01). *NTN1* expression levels were enhanced (*p* < 0.01) under hypoxia and by inflammatory factors in both adipocytes and macrophages. Adipocyte-conditioned media strongly upregulated (*p* < 0.001) the mRNA levels of *NTN1* in macrophages. The treatment of adipocytes with NTN-1 promoted the upregulation (*p* < 0.05) of pro-inflammatory and chemotactic molecules as well as its receptor *NEO1*. Collectively, these findings suggest that NTN-1 regulates VAT chronic inflammation and insulin resistance in obesity.

## 1. Introduction

Obesity constitutes one of the most prevalent diseases reaching epidemic proportions and is becoming a leading global health threat [1,2]. Excess adipose tissue (AT) prompts not only physical and mental impairment, but also increased risk of developing non-communicable comorbidities, including cardiovascular diseases, insulin resistance (IR), type 2 diabetes (T2D), respiratory failure, reduced fertility, and certain types of cancers, therefore shortening lifespans [3,4]. AT-chronic inflammation constitutes a key factor in the onset and progression of obesity-associated metabolic alterations, with the infiltration of monocytes from the bloodstream contributing to its dysregulation and favoring local and systemic inflammation [5]. In this context, the release of chemotactic pro-inflammatory cytokines, including tumor necrosis factor (TNF)-α, C-reactive protein (CRP), interleukin (IL)-1β, IL-6, IL-8, and monocyte chemoattractant protein (MCP)-1 has been extensively studied in the context of obesity-associated chronic inflammation [6,7].

Netrin-1 (NTN-1) is a secreted laminin-like protein member of the neuronal guidance proteins with a crucial function in the regulation of inflammation due to its effects on cell migration [8,9]. NTN-1 exerts chemoattractive or chemorepulsive functions depending on its receptors, including neogenin-1 (NEO-1), deleted in colorectal carcinomas (DCC), A2B receptor (A2BAR), uncoordinated-5 homolog family members (UNC5A, UNC5B, UNC5C, and UNC5D), CD146, and integrin subunits [8]. The involvement of NTN-1 in the regulation of multiple inflammation-related pathologies including cardiovascular and hepatic diseases, cancer and obesity has been widely demonstrated [8,10,11,12]. Interestingly, NTN-1 is highly expressed in the AT from mice and humans with obesity, promoting the accumulation of macrophages and their switch to the M1 phenotype, and thus resulting in increased inflammation and metabolic alterations [13,14]. In this line, Schlegel et al. elegantly described that the silencing of *Ntn1* in mouse monocytes and macrophages was followed by an advanced resolution of inflammation and plaque regression [11]. Moreover, the specific myeloid deletion of *Ntn1* halved the resident AT macrophages of high-fat diet (HFD)-fed mice, suggesting that during obesity, NTN-1 reprograms the macrophage phenotype [14]. Consistent with the attributed pro-inflammatory properties, *Ntn1* levels have been positively correlated with the expression of *Mcp1*, *Tnf*, *Unc5b*, and leptin (*Lep*) in adipocytes and macrophages treated with IL-20, a pro-inflammatory cytokine involved in adipogenesis as well as in macrophage dysregulation in obesity [15]. Moreover, hypoxia-inducible factor 1-α (HIF-1α) induced *NTN1* and its receptor *UNC5B* in macrophages from atherosclerotic plaques, resulting in an impaired migratory capacity of macrophages, which was restored after HIF-1α inhibition [16]. Opposite to the classical view that NTN-1 exerts adverse effects due to macrophage retention in the inflamed tissue [17], other studies have reported that NTN-1 favors the resolution of inflammation [18,19,20]. NTN-1 exhibits contradictory actions that relate to its different functions depending on its relative expression, concentration, receptor types, cell sources and tissues, inflammatory states, and diseases [8].

Given the pleiotropic functions of NTN-1, more studies are required to elucidate the contribution of NTN-1 in VAT inflammation. We hypothesized that NTN-1 is mainly produced by immune cells constituting the stromal vascular fraction cells (SVFC) from VAT with an essential role in the infiltration of macrophages through its receptor NEO-1 perpetuating the inflammatory state in obesity. Therefore, our first objective was to study whether obesity and its associated pathology T2D influence NTN-1 and NEO-1 circulating levels and to analyze the effect of weight loss on their plasma levels. As secondary objectives, we aimed to (i) determine the gene expression levels of *NTN1* and *NEO1* in actively metabolic tissues [VAT, liver, and peripheral blood mononuclear cells (PBMC)] from lean (LN) volunteers and patients with obesity (OB); (ii) analyze the regulation of *NTN1* and *NEO1* by hypoxia and different inflammation-related mediators in human visceral adipocytes and in THP-1-derived macrophages and finally, (iii) to disentangle the effects of NTN-1 in inflammation and the remodeling of the extracellular matrix (ECM) in human adipocytes.

## 2. Materials and Methods

### 2.1. Patient Selection

#### 2.1.1. Study Population

Circulating levels of NTN-1 and NEO-1 were determined in a case-control study with 91 samples (28 men and 63 women) obtained from healthy LN volunteers (*n* = 18) or patients with OB (*n* = 73) at the Clínica Universidad de Navarra. The global inclusion criteria used were 18–65-year-old males and females, body mass index (BMI) between 18.5–24.9 kg/m^2^ for LN subjects and BMI ≥ 30.0 kg/m^2^ for volunteers with OB, absence of psychiatric pathology, and written informed consent for participation in the study. The exclusion criteria were severe systemic disease not related to obesity, infectious/inflammatory diseases, cancer or severe nephropathy, pharmacological treatments, pregnancy or lactation, and people whose freedom is under legal or administrative requirement. According to the Expert Committee on Diabetes Diagnosis and Classification, patients with OB were subclassified into two groups: with normoglycaemia (NG, *n* = 32) or with T2D (*n* = 41) [21]. Subjects with T2D were not taking any medications that could interfere with endogenous insulin levels and did not have a long history of diabetes.

#### 2.1.2. Anthropometric Measurements

Body weight and height were measured with a digital scale to the nearest 0.1 kg and with a Harpenden stadiometer to the nearest 0.1 cm, respectively (Holtain Ltd., Crymych, UK). BMI was calculated dividing kilograms of weight by the square of the height in meters (BMI = weight [kg]/height [m^2^]). To measure the waist circumference, a non-elastic tape was placed between the iliac crest and the rib cage. Air displacement plethysmography (Bod-Pod^®^, Life Measurements, Concord, CA, USA) was used to estimate body fat (BF). Blood pressure was measured after 5 min of rest in a semi-sitting position with a sphygmomanometer. Blood pressure was measured in the upper right arm at least 3 times to calculate the mean of the analyses.

#### 2.1.3. Weight Loss Achieved by Bariatric Surgery and Dietary Treatment

To compare the impact of weight loss achieved by caloric restriction or Roux-en-Y gastric bypass (RYGB) on NTN-1 and NEO-1 plasma concentrations, volunteers submitted to either RYGB (*n* = 23) or a conventional dietary treatment (*n* = 14) (both evaluated after 9 months) were used. Preoperatively, a complete dietetic history was performed to determine potential nutritional deficiencies, and to optimize the micronutrient status before surgery. Interventions were carried out by the same bariatric team. Postoperative interventions followed the hospital bariatric unit protocol. Patients were prescribed oral vitamin and micronutrient supplements to compensate for their possible reduced intake and absorption as previously described [22]. Conventional dietary treatments consisted of a personalized diet prescribed by a physician in collaboration with a dietitian with planned regular follow-up visits to ensure a daily caloric deficit of 500–1000 kcal/d. The hypocaloric diet allows a steady and safe weight loss of 0.5 to 1.0 kg/week.

#### 2.1.4. Surgical Procedures and Tissue Collection

Following a laparoscopic procedure, VAT samples from patients undergoing Nissen fundoplication (for hiatal hernia repair in LN volunteers, *n* = 7) or RYGB (for treatment of severe obesity, *n* = 56) were collected. During bariatric surgery, blood samples, and an intraoperative hepatic biopsy for the histological diagnosis of their liver state and to examine, the gene expression levels of *NTN1* and *NEO1* were obtained. Hepatic biopsies were omitted in LN subjects due to the lack of clinical justification. The diagnosis of nonalcoholic fatty liver disease was established by the anatomo-pathological analysis of liver biopsies using the criteria of Kleiner and Brunt [23]. The tissue samples were stored at −80 °C for further analysis.

#### 2.1.5. Ethical Considerations

The study protocol was designed according to the guidelines of the Declaration of Helsinki and was approved by the Clínica Universidad de Navarra’s Ethical Committee responsible for research (2019.089). Written informed consent was obtained from all the participants.

### 2.2. Analytical Measurements

Venipuncture was conducted following an overnight fast to obtain plasma and serum samples to determine the metabolic and inflammatory profiles of LN volunteers and patients with OB, as well as NTN-1 and NEO-1 circulating levels. An automated analyzer was used to perform glucose analysis (Hitachi Modular P800, Roche, Basel, Switzerland), and insulin was measured with an enzyme amplified chemiluminescence assay (IMMULITE^®^, Diagnostic Products Corp., Los Angeles, CA, USA). Biochemical assays to analyze the glucidic, lipidic, hepatic, and inflammatory profiles were performed as previously reported [24]. Uric acid, alanine aminotransferase (ALT), aspartate aminotransferase (AST), and γ-glutamyltransferase (γ-GT) were measured enzymatically (Hitachi Modular P800, Roche). C-reactive protein (CRP), fibrinogen, and antigen von Willebrand factor (vWF) concentrations were determined as previously described [24]. A double antibody RIA technique (Linco Research, Inc., St. Charles, MO, USA) was used to analyze leptin levels [25]. Inter- and intra-assay coefficients of variation were 4.5% and 5.0%, respectively. Circulating levels of NTN-1 and NEO-1 were measured by ELISA kits (CUSABIO, College Park, MD, USA) with intra- and inter-assay coefficients of variation being 8.0% and 10.0%, respectively.

### 2.3. Analysis of Gene Expression Levels

Since different tissues contribute to obesity-associated inflammation, we investigated the mRNA levels of *NTN1* and *NEO1* in a subgroup of volunteers in VAT (*n* = 62) and liver (*n* = 42) samples, as well as in PBMC (*n* = 61). In addition, to determine which cell type within VAT contributed to the expression of *NTN1* and *NEO1*, their gene expression levels were also analyzed in adipocytes and SVFC. Total RNA was isolated using an Ultra-Turrax^®^ T25 basic (IKA-Werke GmbH, Staugen, Germany) and TRIzol^TM^ Reagent (Invitrogen, Carlsbad, CA, USA) for liver, PBMCs, SVFC, and THP-1-derived macrophages, as well as QIAzol^®^ Reagent (Qiagen, Hilden, Germany) for VAT and adipocytes. Samples were purified with the RNeasy Mini kit (Qiagen) and treated with DNase I (Qiagen) to remove genomic DNA as previously reported [26]. For the synthesis of the first cDNA strand, 3 µg of total RNA were reverse transcribed in a final volume of 60 µL using random hexamers (Roche) as primers and 300 units of M-MLV reverse transcriptase (Invitrogen). Transcription levels of adiponectin (*ADIPOQ*), apoptosis-associated speck-like protein containing a CARD (*ASC*), *DCC*, *IL1A*, *IL1B*, *IL6*, *IL32*, *IL36*, matrix metalloproteinase (*MMP*)-2, -9, *MCP1/CCL2*, *NEO1*, NLR family pyrin domain containing 3 (*NLRP3*), nucleotide-binding oligomerization domain containing protein 2 (*NOD2*), *NTN1*, semaphoring 3E (*SEMA3E*), osteopontin (*SPP1*), transforming growth factor-β (*TGFB*), tenascin C (*TNC*), *TNF,* and *UNC5B* were quantified by Real-Time PCR (7300 Real Time PCR System, Applied Biosystem, Foster City, CA, USA). Primers and probes (Table S1) were designed using Primer Express 2.0 software (Applied Biosystems) acquired from Genosys (Merck, Darmstadt, Germany). To avoid genomic DNA amplification, the probes covered the ends of two exons. The cDNA was amplified as previously described [26].

### 2.4. Histological Analysis of NTN-1 and NEO-1

Immunohistochemistry was performed as previously described to evaluate the cellular localization of NTN-1 and NEO-1 in VAT [27]. Briefly, VAT sections (6 µm) from OB-NG and OB-T2D patients were formalin-fixed, dewaxed in xylene and rehydrated in decreasing concentrations of ethanol. Sections were incubated overnight at 4 °C with a goat anti-human NTN-1 monoclonal antibody (R&D Systems, Minneapolis, USA) diluted 1:100 in Tris buffered saline (TBS, Merck) and a rabbit anti-human NEO-1 monoclonal antibody (R&D Systems) diluted 1:50 on TBS. After rinsing the slides with TBS, they were incubated for 1 h at room temperature with anti-goat and anti-rabbit secondary antibodies conjugated with Dako^TM^ Real^TM^ EnVision^TM^ horseradish peroxidase (DakoCytomation, Glostrup, Denmark). Sections were dehydrated in increasing concentrations of ethanol, mounted with DePeX mounting medium (Serva, Heidelberg, Germany) and observed under a Zeiss Axiovert CFL light microscope (Zeiss, Göttingen, Germany) at 20X. A negative control slide was included in which the primary antibody was replaced by TBS to assess nonspecific staining.

### 2.5. Adipocyte and Monocyte Cultures

SVFC from patients with OB were isolated from VAT and differentiated to adipocytes [27]. The adipocyte conditioned media (ACM) was prepared by collecting the supernatant of differentiated adipocytes and further centrifuged and diluted (20% and 40%). ACM was used to evaluate the effects of adipocyte secretion on the expression of *NTN1* and *NEO1* mRNA in monocyte-derived macrophages. Human monocytic leukemia cells (THP-1) (TIB-202™, ATCC, Middlesex, UK) were cultured in suspension with RPMI 1640 medium supplemented with 10% fetal bovine serum, 100 U/mL penicillin, 100 µg/mL streptomycin, 1 mM L-glutamine, 50 µM β-mercaptoethanol, and 1 mM sodium pyruvate. THP-1 monocytes were treated with 25 ng/mL phorbol 12-myristate 13 acetate (PMA, Merck) for 48 h to polarize macrophages towards the pro-inflammatory M1 phenotype. 

To understand the mechanisms underlying *NTN1* and *NEO1* expression during obesity, we treated human visceral adipocytes and THP-1-derived macrophages with exogenous and endogenous inflammation-related factors. Differentiated adipocytes and macrophages were serum starved for 24 h and 2 h, respectively, and then treated with increasing concentrations of lipopolysaccharide (LPS) (Merck), TNF-α (Merck), cobalt chloride (CoCl_2_) (Merck), IL-4 (R&D Systems), IL-13 (R&D Systems), and kallistatin (R&D Systems) for 24 h. Since a dynamic interaction between adipocytes and macrophages exists, we evaluated the influence of the ACM obtained from subjects with OB on *NTN1* and *NEO1* gene expression levels in THP-1-derived macrophages. To confirm whether NTN-1 induces the expression of genes promoting an inflammatory response and chemotaxis, we stimulated adipocytes with increasing concentrations of NTN-1 (R&D Systems) for 24 h.

### 2.6. Data and Statistical Analysis

The data is presented as mean ± SEM. Due to their non-normal distribution, mRNA levels and CRP concentrations were logarithmically transformed for statistical analysis. The G*Power 3.1.9.4 program (Franz Faul, University of Kiel, Germany) was used to calculate the sample size using the mean and the standard deviation of the preliminary data obtained in our own experience [22]. One-way ANOVA, followed by Tukey’s or Dunnett’s *post hoc* tests and two-tailed paired or unpaired Student’s *t* tests, were used to examine differences between groups. Pearson’s correlation coefficient (r) was used to analyze the association between variables. The statistical analysis was performed with SPSS/Windows version 15.0 statistical package (SPSS, Chicago, IL, USA) and graphs were created with GraphPad Prism version 8.3 (GraphPad Software, Inc., San Diego, CA, USA). A *p* value < 0.05 was considered statistically significant.

## 3. Results

### 3.1. Clinical Characteristics of the Study Population

Clinical features of the study population are shown in Table 1. No age differences between groups were observed. Patients with OB showed increased (*p* < 0.01) mean systolic and diastolic blood pressure compared to LN subjects. As expected, anthropometric characteristics (BMI, BF, waist and hip circumference, and waist-to-hip ratio) were higher (*p* < 0.01) in both groups with OB compared to LN subjects. Glycaemia, insulin levels, and HOMA were increased (*p* < 0.01) in OB-T2D compared to OB-NG and LN individuals, whereas QUICKI index was decreased (*p* < 0.01). Patients with OB also exhibited higher leptin (*p* < 0.01) and triglyceride concentrations (*p* < 0.01) as well as lower HDL cholesterol levels (*p* < 0.05) compared to LN volunteers. All inflammatory factors were significantly increased (*p* < 0.01) in both groups of individuals with OB.

### 3.2. Circulating Levels of NTN-1 Are Increased in Obesity and T2D and Decreased after Conventional Weight Loss

As shown in Table 1, circulating levels of NTN-1 increased (*p* < 0.001) in OB-NG and OB-T2D patients compared to LN volunteers while no differences in NEO-1 circulating levels were detected. In this sense, a strong association between NTN-1 circulating levels and all anthropometric parameters as well as with leptin and fibrinogen concentrations was found (Table 2). Interestingly, NTN-1 was positively correlated with insulin and HOMA index and negatively with QUICKI (Table 2).

In the 9-month follow-up study after diet or surgery, patients improved anthropometric and metabolic parameters, with differences being more remarkable after surgery (Table 3).

**Figure 1:**
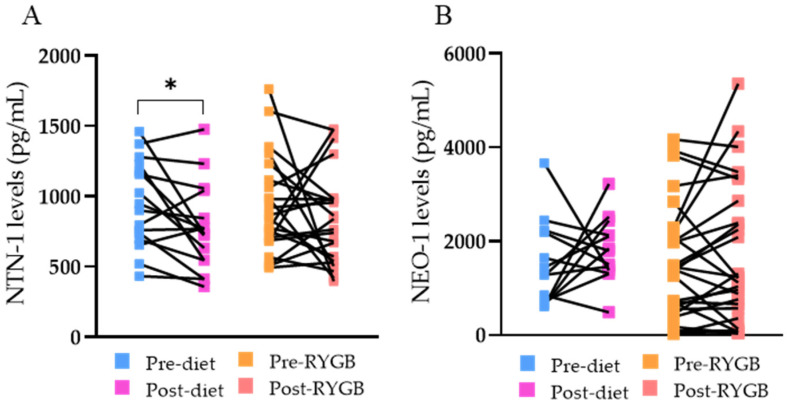
(**A**) Circulating levels of netrin (NTN)-1 before and after weight loss achieved by a conventional diet (*n* = 16) or Roux-en-Y gastric bypass (RYGB) (*n* = 22). (**B**) Circulating levels of neogenin (NEO)-1 before and after weight loss achieved by a conventional diet (*n* = 13) or Roux-en-Y gastric bypass (RYGB) (*n* = 28). Differences between groups were analyzed by unpaired Student’s *t*-test. * *p* < 0.05.

Although no changes were observed after surgery, circulating concentrations of NTN-1 decreased (*p* = 0.023) after diet-induced weight loss (Figure 1A). No differences in circulating levels of NEO-1 were observed after bariatric surgery or a hypocaloric diet (Figure 1B). Noteworthy, reduced NTN-1 concentrations after caloric restriction were significantly associated with decreased glucose (r = 0.78; *p* = 0.005) and insulin (r = 0.68; *p* = 0.043) levels.

Our data suggest that increased circulating NTN-1 levels in obesity decrease after diet-induced weight loss being also associated with a reduction in IR.

### 3.3. Upregulated Gene Expression Levels of NTN1 in Visceral Adipose Tissue in Obesity

**Figure 2:**
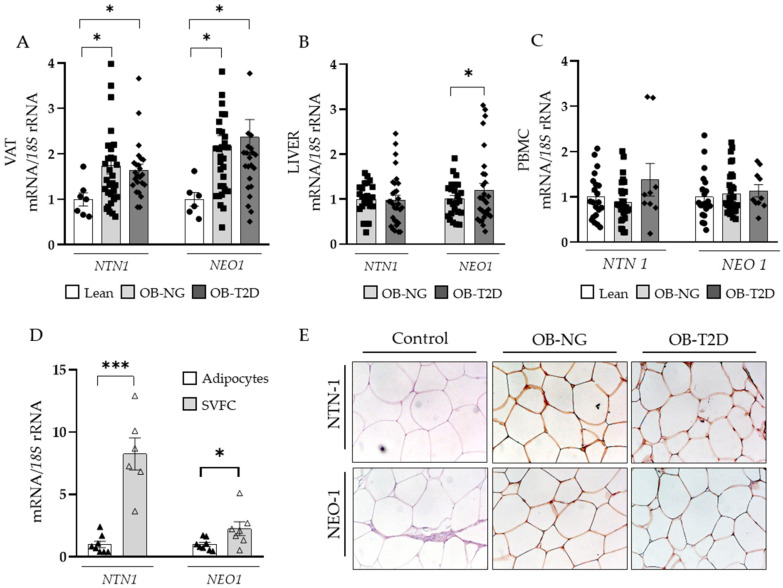
Gene expression levels of *NTN1* and *NEO1* in (**A**) visceral adipose tissue (VAT, LN *n* = 7, OB-NG *n* = 31, OB-T2D *n* = 24), (**B**) liver (OB-NG *n* = 18, OB-T2D *n*= 24), and (**C**) peripheral blood mononuclear cells (PBMC, LN *n* = 21, OB-NG *n* = 30, OB-T2D *n* = 10) from lean volunteers (LN), patients with obesity and normoglycaemia (OB-NG) and patients with obesity and T2D (OB-T2D). (**D**) mRNA levels of *NTN1* and *NEO1* in adipocytes (*n* = 8) and stromal vascular fraction cells (SVFC *n* = 6–7) and (**E**) immunohistochemical detection of NTN-1 and NEO-1 in VAT obtained from patients with OB and with and without T2D (magnification 20X). Bars represent the mean ± SEM. Differences between groups were analyzed by one-way ANOVA followed by Tukey’s *post hoc* tests or unpaired Student’s t-test, where appropriate. * *p* < 0.05 and *** *p* < 0.001.

Increased gene expression levels (*p* < 0.05) of *NTN1* and *NEO1* in VAT in both OB groups were found (Figure 2A). In this sense, *NTN1* and *NEO1* mRNA levels were significantly associated between them (r = 0.47; *p* < 0.01) as well as with BF (*p* < 0.05) (Figure S1). Results also showed increased mRNA levels (*p* < 0.05) of *NEO1* in the liver of patients with OB and T2D (Figure 2B). Although a tendency for an increased expression of *NTN1* and *NEO1* in PBMC from patients with T2D was found, differences were not statistically significant (Figure 2C). In comparison to adipocytes, SVFC from patients with OB exhibited higher mRNA levels of *NTN1* (*p* < 0.001) and *NEO1* (*p* < 0.05) (Figure 2D). These findings were further confirmed by immunohistochemistry (Figure 2E). 

Gene expression levels of *NTN1* were increased in the VAT from patients with OB, probably as a result of the increased infiltrating immune cells.

### 3.4. NTN1 and NEO1 Are Associated with ECM Remodelling and Inflammation in VAT

Given the role of NTN-1 and NEO-1 in cell migration [9], we analyzed their association with relevant genes related with ECM remodeling in VAT. Patients with OB showed increased mRNA expression levels (*p* < 0.05) of the ECM genes *MMP9*, *TNC,* and *TGFB* with the latter being also upregulated (*p* < 0.001) in T2D patients compared to NG subjects (Table 4). A positive correlation of the gene expression levels of *NTN1* and *NEO1* with *MMP2* (r = 0.35 and r = 0.42, respectively; *p* < 0.01) and *TGFB* (r = 0.32 and r = 0.30, respectively; *p* < 0.05) was found. *NEO1* gene expression levels were also associated to *TNC* (r = 0.45; *p* < 0.01) and *TNF* (r = 0.32; *p* < 0.01).

Next, we assessed the association of NTN-1 and NEO-1 with key pro-inflammatory molecules implicated in the chronic inflammation of VAT (Table 4). Both groups of patients with OB showed increased mRNA levels (*p* < 0.01) of *ASC*, *IL1B*, *IL6*, *NLRP3,* and *NOD2,* compared to LN volunteers in the VAT. Furthermore, *IL1B* and *IL6* mRNA levels were also increased (*p* < 0.05) in patients with T2D compared to NG subjects. As expected, gene expression levels of *ADIPOQ* were significantly downregulated (*p* < 0.01) in the VAT from both groups of patients with OB compared to LN volunteers. In addition, the gene expression levels of *NTN1* and *NEO1* were associated with *ASC* (r = 0.43 and r = 0.37; *p* < 0.01) and *NTN1* expression with *NLRP3* (r = 0.56; *p* < 0.01), *NOD2* (r = 0.55; *p* < 0.01), and *IL1A* (r = 0.37; *p* < 0.01) mRNA levels.

### 3.5. Inflammation-Related Factors Regulate NTN1 Expression Levels in Human Visceral Adipocytes and THP-1-Derived Macrophages

**Figure 3:**
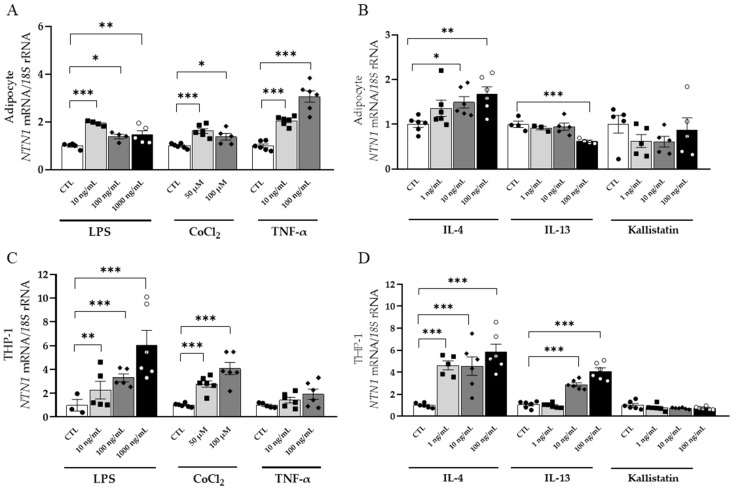
(**A**,**C**) Effect of the pro-inflammatory factors LPS, CoCl_2_ and TNF-α and (**B**,**D**) the anti-inflammatory mediators IL-4, IL-13, and kallistatin on *NTN1* gene expression levels in human differentiated visceral adipocytes and monocyte-derived macrophages. Gene expression levels in unstimulated cells were assumed to be 1. Differences between groups were analyzed by one-way ANOVA followed by Dunnett’s *post hoc* tests. * *p* < 0.05, ** *p* < 0.01, and *** *p* < 0.001. CTL, control.

*NTN1* mRNA levels were strongly upregulated (*p* < 0.001) by LPS and CoCl_2_ in visceral adipocytes and THP-1 cells while *NEO1* levels remained unchanged (Figure 3A,C and Figure S1). In turn, stimulation with TNF-α highly increased (*p* < 0.001) the expression of *NTN1* in adipocytes with no differences found in macrophages (Figure 3A,C). A significant downregulation (*p* < 0.001) of *NTN1* was observed after IL-13 treatment in adipocytes and its mRNA levels significantly increased in THP-1 (*p* < 0.001) (Figure 3B,D). Unexpectedly, IL-4 boosted *NTN1* expression in adipocytes and macrophages (*p* < 0.01 and *p* < 0.001, respectively) (Figure 3B,D). After the stimulation with kallistatin, an upregulation in the expression of *NEO1* (*p* < 0.001), but not *NTN1*, was observed in adipocytes and no changes were found in macrophages (Figure S2).

**Figure 4:**
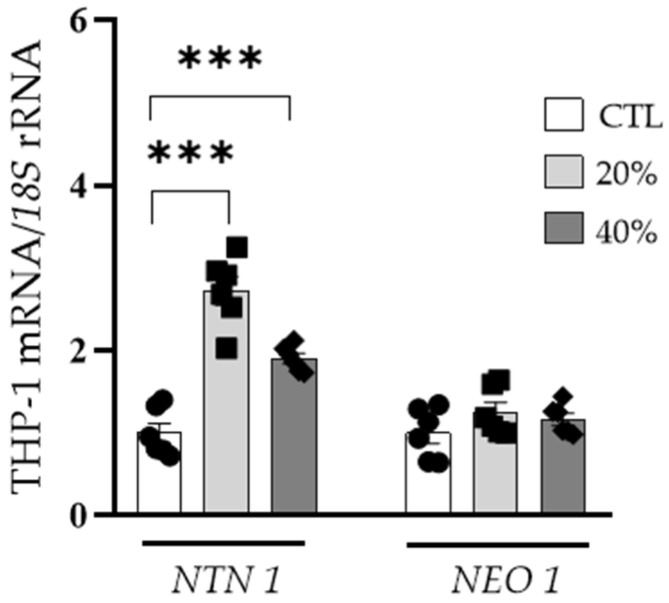
Effect of adipocyte conditioned medium (20% and 40%) on *NTN1* and *NEO1* gene expression levels in monocyte-derived macrophages. Gene expression levels in unstimulated cells were assumed to be 1. Differences between groups were analyzed by one-way ANOVA followed by Dunnett’s *post hoc* tests. *** *p* < 0.001. CTL, control.

Interestingly, we found increased mRNA levels of *NTN1* (*p* < 0.01) in THP-1-derived macrophages treated with 20% and 40% ACM, but no differences in *NEO1* expression levels were detected (Figure 4).

The treatment of human adipocytes and macrophages with inflammatory molecules exhibited increased *NTN1* expression levels being also upregulated in macrophages treated with the adipocyte-derived secretome obtained from patients with OB.

### 3.6. Effects of NTN-1 in the Gene Expression of Inflammation- and Chemotactic-Related Factors in Visceral Adipocytes

**Figure 5:**
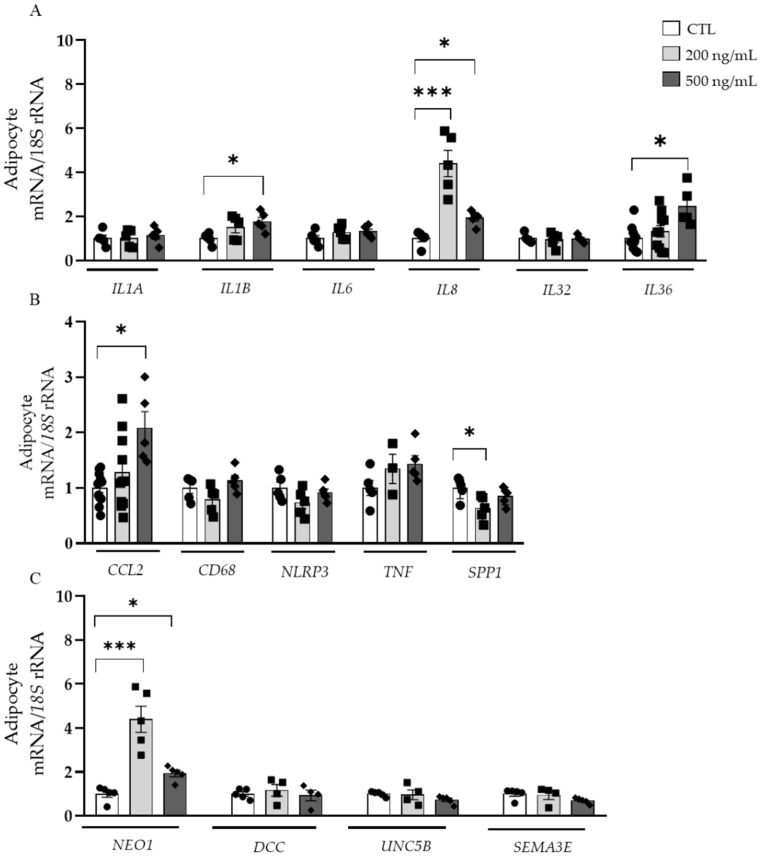
Impact of NTN-1 on the gene expression levels of (**A**) inflammatory interleukins, (**B**) acute inflammatory markers and chemotactic cytokines, and (**C**) NTN-1 receptors and *SEMA3E* in human visceral adipocytes. Gene expression levels in unstimulated cells were assumed to be 1. Differences between groups were analyzed by one-way ANOVA followed by Dunnett’s *post hoc* tests. * *p* < 0.05 and *** *p* < 0.001. *CCL2*, monocyte chemoattractant protein-1; *DCC*, deleted in colorectal cancer; *IL*, interleukin; *NEO1*, neogenin-1; *NLRP3*, NLR family pyrin domain containing 3; *NTN1*, netrin-1; *SEMA3E*, semaphorin 3E; *SPP1*, osteopontin; *TGFB*, transforming growth factor-β; *TNF*, tumor necrosis factor-α; *UNC5B,* UNC-5 netrin receptor B.

After the treatment of human visceral adipocytes with NTN-1, an upregulation of *IL1B* (*p* < 0.05), *IL8* (*p* < 0.001), *IL36* (*p* < 0.05), and *CCL2* (*p* < 0.05) together with a downregulation of *SPP1* (*p* < 0.05) were observed (Figure 5A,B). No differences in *IL1A*, *IL6*, *IL32*, *CD68*, *NLRP3,* and *TNF* mRNA levels were found. Since NTN-1 has been described to exert its immunomodulatory functions through different receptors, we evaluated the effect of NTN-1 in their expression levels. Although the expression of *DCC* and *UNC5B* remained unchanged, a strong upregulation (*p* < 0.001) of *NEO1* after the treatment with NTN-1 was detected (Figure 5C). Due to the chemoattractant role that SEMA3E can exert on macrophages in the VAT [28], we explored whether the presence of NTN-1 modulated its mRNA levels, but no differences were found (Figure 5C).

## 4. Discussion

**Figure 6:**
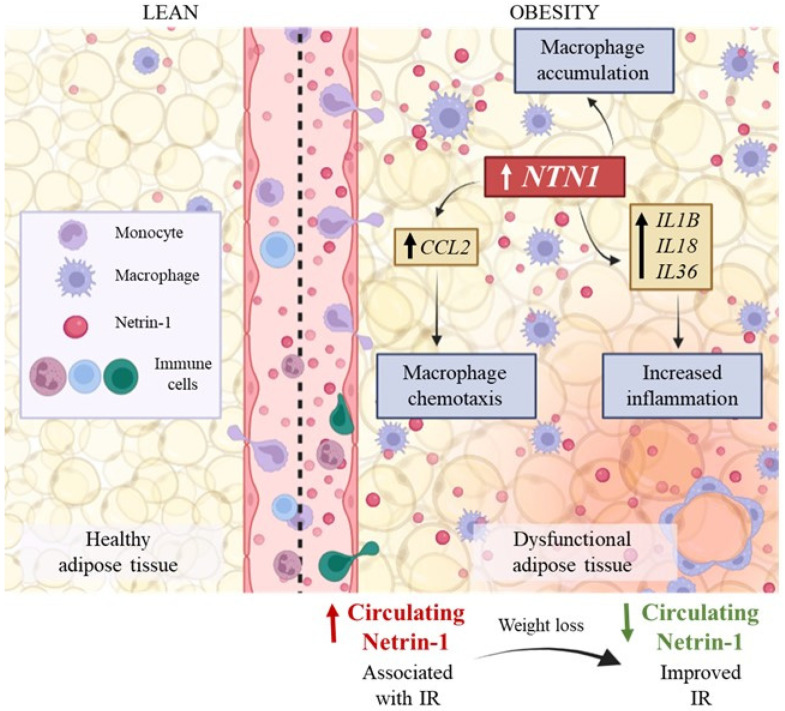
Dysfunctional AT in obesity is characterized by impaired angiogenesis, local hypoxia, and fibrosis, as well as chronic and unresolved inflammation with a special upregulation in the expression of pro-inflammatory adipokines. These metabolic alterations and the limitation in adipose tissue expandability are strongly associated to the onset of obesity-related comorbidities. In this context, the increased expression levels of *NTN1* in visceral AT, mainly due to the SVFC, may be favoring inflammation promoting macrophage chemotaxis by the upregulation of *CCL2,* as well as by the induction of *IL1B*, *IL18,* and *IL36*. Moreover, the increased circulating levels of NTN-1 in obesity were associated with IR, and the decrease of NTN-1 after caloric restriction were positively correlated with differences in glucose and insulin levels, suggesting the role of NTN-1 in the development of IR. AT, adipose tissue; *CCL2*, C-C motif chemokine ligand 2; *IL*, interleukin; IR, insulin resistance; *NTN1*, netrin-1; SVFC, stromovascular fraction cells.

Although NTN-1 plays crucial functions in inflammatory diseases, little is known about its regulation and roles in the obesity-associated VAT inflammation. In the present study, we found that it: (i) increased circulating NTN-1 levels in obesity decrease after diet-induced weight loss being also associated with a reduction in IR; (ii) gene expression levels of NTN1 and NEO1 were upregulated in VAT from patients with OB being the highest expression in the SVFC, (iii) human adipocytes and macrophages stimulated with inflammatory molecules exhibited increased NTN1 levels, (iv) NTN1 was upregulated in macrophages treated with ACM from patients with OB, reinforcing that adipocytes could trigger immune cells infiltration, retention and activation, and (v) the treatment of adipocytes with NTN-1 promoted the upregulation of proinflammatory and chemotactic molecules as well as its receptor NEO1 (Figure 6).

We showed increased circulating levels of NTN-1 in patients with OB and with T2D. Reportedly, patients with impaired glucose tolerance and T2D exhibited higher circulating levels of NTN-1 being also positively correlated with IR [29,30] (Figure 6). Opposite results have been also described, with levels of NTN-1 being decreased in patients with metabolic disorders including obesity and T2D [12,31]. Our findings showed a positive correlation of NTN-1 with insulin levels and HOMA and a negative association with QUICKI in line with previous studies [30]. Consistent to our results, loss of Ntn1 expression in AT macrophages lead to improved maintenance of glucose homeostasis and insulin sensitivity during obesity [13]. However, a negative association with markers of IR, including HbA1c, fasting glucose, or HOMA-IR have been also found [32]. The different commercial netrin-1 ELISA kits used in the studies or the distinct characteristics of study population may explain these inconsistent findings. In our study, subjects with T2D were drug-naïve, reducing the effects of anti-T2D therapies on the circulating levels of NTN-1.

Interestingly, circulating NTN-1 concentrations decreased after weight loss achieved by caloric restriction, probably influenced by metabolic and hormonal changes, as occurs with other inflammatory markers such as plasminogen activator inhibitor-1, CRP, serum amyloid A or chitinase 3-like 1 (YKL-40) [33,34]. NTN-1 levels also tended to decrease after 9 months of RYGB, but differences did not reach statistical significance, possibly due to the inflammatory state intrinsically induced by surgery during the first months after the intervention [35,36]. Importantly, changes in NTN-1 concentrations after caloric restriction were positively correlated with differences in glucose and insulin levels, which strengthens the role of NTN-1 in the development of IR. In this sense, circulating levels of NTN-1 could reflect the degree of insulin sensitivity and potentially serve as a biomarker to indicate the severity of obesity (Figure 6).

We found higher NTN1 and NEO1 expression levels in the VAT from patients with OB and with T2D. In parallel, Ramkhelawon et al. also observed higher Ntn1 in the epididymal AT from diet-induced obese mice compared to lean chow-fed mice [13]. Specifically, we observed that *NTN1 and NEO1* mRNA levels were increased in SVFC compared to adipocytes, indicating that immune cells may be the main responsible of NTN-1 release in VAT. Consistent with our findings, immunofluorescence staining in the EWAT from HFD-fed mice revealed that Ntn-1 and its receptor Unc5b reacted in crown-like structures where macrophages were colocalized for the F4/80 antigen [13]. NTN-1 was also highly expressed by macrophages in atherosclerotic plaques leading to the accumulation of macrophages and disease progression [17]. In addition, macrophage migration was favored in Ntn1 knockout mice, which translates into a marked role of Ntn-1 in macrophage retention. The targeted deletion of Ntn1 in macrophages reprogramed the phenotype of AT macrophages in obesity, promoting reduced adipose inflammation and a functional enhancement of lipid flow in AT that drove an improved metabolic function [14]. In this sense, we suggest that the pro-inflammatory profile acquired by SVFC during obesity may promote the retention of macrophages in VAT by the release of NTN-1, favoring a cycle of inflammatory cascades in the VAT (Figure 6). According to the recently described potential link between hepatic inflammation and liver disease progression through netrin-1 [10], we found an upregulation of its receptor NEO1 in the liver from patients with T2D.

The chronic inflammation of the AT during obesity can lead to non-resolving inflammation and the onset of metabolic diseases [2,5,37]. In this line, we found upregulated levels of the inflammasome complex NLRP3, its adaptive protein ASC, and its effector protein IL1B together with the inflammatory cytokines IL16, IL8, and IL1A in the VAT from patients with OB. In addition, we showed a positive correlation between NTN1 and the key pro-inflammatory factors ASC, IL1A, NLPR3, and NOD2, strengthening the involvement of NTN-1 in VAT chronic inflammation. Given the numerous studies demonstrating inflammation as a main contributor to obesity-related IR and since NTN-1 plays a critical role in VAT inflammation, these findings point to NTN-1 as an important component in the pathogenesis of IR [12,13,38]. However, other studies have described NTN-1 as an anti-inflammatory factor, especially in diseases characterized by acute inflammatory reactions and in highly vascularized tissues such as the lungs or the heart [9,39,40,41]. This hypothesis suggests that NTN-1 may exerts pro-inflammatory actions in poorly vascularized tissues, such as the AT as well as during chronic inflammatory states, as occurs in obesity.

To study the expression patterns of NTN1 and NEO1 in the VAT during obesity, we treated adipocytes and monocytes with different inflammation-associated factors. Similar to the increase in NTN1 expression levels observed in adipocytes and macrophages after the stimulation with LPS and TNF-α, Berg et al. found NTN1 as the main upregulated neuronal guidance protein in macrophages treated with LPS [42]. In his line, Ntn1 was increased in 3T3-L1 adipocytes treated with TNF-α and IL-6. Other inflammatory insults, such as palmitate, have been shown to induce the expression of Ntn1 via a NF-*κ*B-dependent pathway [13]. We also showed that NTN1 mRNA levels increased after mimicking hypoxic conditions with CoCl_2_. An increase in Ntn1 mRNA levels in macrophages under hypoxic conditions has previously been described [16]. Furthermore, Hif1a knockout mice were unable to secrete Ntn-1 during LPS-induced lung injury [42]. In this line, HIF-1α has been proposed as a key transcriptional inducer of NTN-1 signaling. The opposite expression profile in adipocytes and macrophages observed after the stimulation with IL-13 may be attributed to the different function these cells fulfil in the VAT. Moreover, NEO1 mRNA levels were downregulated in macrophages after IL-13 treatment, possibly as a compensatory mechanism to block the signaling of NTN-1 into the cell. The anti-inflammatory kallistatin exerted a role in adipocytes increasing the mRNA levels of NEO1 likely in an attempt to decrease NTN-1 levels in the extracellular space and reduce inflammation [43]. To simulate the environment found in the VAT during obesity, we studied the effect of adipocyte-derived factors obtained from patients with OB on NTN1 and NEO1 expression levels in macrophages. A significant increase of NTN1 expression levels was observed, supporting a crosstalk between adipocytes and macrophages, and suggesting that dysregulated adipocytes in obesity may increase the expression of NTN1 in AT macrophages triggering monocyte infiltration and retention in VAT. In turn, the treatment of T3T-L1 adipocytes with the conditioned media from bone marrow-derived macrophages previously stimulated with free fatty acids strongly induced the expression of Ntn1 [13].

Depending on the receptor NTN-1 binds to in the VAT, it may promote different downstream signaling effects related to migration, macrophage retention or inflammation [8]. Thus, we sought to elucidate the receptors through which signals in the VAT. The upregulation of NEO1 in adipocytes after the treatment with NTN-1 together with the lack of changes in the expression of UNC5B and DCC suggest that NTN-1 mainly signals through NEO-1 in VAT during obesity, being reinforced by the positive correlation found between the expression levels of NTN1 and NEO1 in the VAT. The increase in the expression of CCL2, IL1B, IL8, and IL36 after the treatment with NTN-1 strengthen the role of NTN-1 in macrophage chemotaxis and inflammation in visceral AT (Figure 6). CCL2 is highly expressed in the AT in obesity and the release of MCP-1 from adipocytes constitutes a key signal for monocyte influx [44]. The increased levels of CCL2 found in the presence of NTN-1 agree with previous findings showing that NTN-1 recruits monocytes into the VAT, also potentiating IR [13]. A protective effect on glucose metabolism and IR similar to that observed in Ccr2 knockout mice was confirmed in mice with hematopoietic deletion of Ntn1 [13]. Similarly, binding of NTN-1 to UNC5B in hepatocytes resulted in the expression of the inflammatory cytokines CCL2, CCL3, CCL5, and CCL8, and also in the accumulation of Ly6C^+^ macrophages in the liver [10]. In this regard, the humanized anti-netrin capture antibody NP137 (US Clinical Trials #02977195) displayed an anti-inflammatory activity in chronic liver disease, identifying NTN-1 as a significant proinflammatory factor for the development of this pathology [45]. The IL-1 family, mainly IL-1*β*, exerts crucial functions in the AT inflammation during obesity constituting key therapeutic targets to ameliorate the adverse metabolic consequences of obesity [46]. The increased levels of IL1B, IL8 and IL36 found after NTN-1 treatment may impair the function of adipocytes promoting a vicious cycle of pro-inflammatory cascades (Figure 6). Considering the pro-inflammatory nature of osteopontin, we found an unexpected decrease in its expression levels. Future research is required to determine whether the decrease of SPP1 is indicative of a compensatory reaction.

Some potential limitations of our study should be pointed out. First, although the number of subjects in the groups may appear somewhat limited, a detailed characterization of our subjects has been performed and the patients included in the study showed a high homogeneity within groups. Second, the study was performed in white people and would need to be extended to other demographics. Third, it would be important to consider the influence of other metabolic and inflammatory factors as well as different micronutrients in the regulation of NTN1 expression as well as the analysis of the impact of NTN-1 on the expression of additional receptors.

Future studies are required to elucidate the exact role of NTN-1 in monocyte infiltration and accumulation in VAT as well as to assess the impact of its downregulation in the obesity-associated chronic inflammation and IR. The study will direct the research towards other important roles that NTN-1 may play in obesity including the regulation of insulin sensitivity and oxidative stress. Moreover, weight loss may improve obesity-associated IR through affecting the levels of NTN-1. Finally, the regulation of immunometabolism through NTN-1 actions may be critical to our understanding of obesity and metabolic dysfunction constituting an important therapeutic approach.

The increased expression levels of NTN1 in the dysfunctional VAT from patients with OB may be aggravating inflammation by the induction of IL1B, IL18, and IL36 at the same time as favoring macrophage infiltration by the upregulation of CCL2. Moreover, the increased circulating levels of NTN-1 in obesity were associated with IR and the decrease of NTN-1 after caloric restriction was positively correlated with differences in glucose and insulin levels, suggesting the role of NTN-1 in the development of IR (Figure 6).

## 5. Conclusions

In summary, the findings of our case-control study suggest that NTN-1 is an inflammatory factor with increased circulating concentrations in patients with obesity and mainly secreted by the SVFC from the VAT that favors chronic inflammation and IR in obesity.

## Figures and Tables

**Table 1:** Clinical and biochemical characteristics of the subjects included in the study.

	Lean	Obesity with Normoglycaemia	Obesity with Type 2 Diabetes	Reference Interval
n (male, female)	18 (8, 10)	32 (7, 25)	41 (13, 28)	
Age (years)	42 ± 5	40 ± 3	47 ± 2	-
Body mass index (kg/m^2^)	22.3 ± 0.7	43.4 ± 1.6 ***	44.3 ± 1.1 ***	-
Body fat (%)	22.1 ± 1.9	52.6 ± 1.5 ***	49.5 ± 1.2 ***	-
Waist-to-hip ratio	0.80 ± 0.03	0.87 ± 0.02 *	1.00 ± 0.01 ***	-
SBP (mm Hg)	105 ± 2	128 ± 3 ***	138 ± 3 ***^,†^	<135 mm Hg
DBP (mm Hg)	66 ± 2	83 ± 2 **	87 ± 2 ***	<85 mm Hg
Fasting glucose (mg/dL)	88 ± 4	90 ± 2	130 ± 8 ***^,†††^	75–100 mg/dL
2h OGTT glucose (mg/dL)	ND	119 ± 3	167 ± 8 ^†††^	<140 mg/dL
Fasting insulin (µU/mL)	6.9 ± 1.3	16.3 ± 2.0	34.2 ± 7.0 **^,†^	5.0–29.1 µU/mL
2h OGTT insulin (µU/mL)	ND	116 ± 17	154 ± 15 ^†††^	-
HOMA	1.5 ± 0.4	3.7 ± 0.5	8.6 ± 0.8 ***	-
QUICKI	0.375 ± 0.016	0.324 ± 0.006 **	0.291 ± 0.005 ***^,†††^	-
Triglycerides (mg/dL)	65 ± 9	99 ± 8	147 ± 9 ***^,††^	<150 mg/dL
Cholesterol (mg/dL)	168 ± 6	198 ± 8	184 ± 5	<200 mg/dL
LDL-cholesterol (mg/dL)	95 ± 8	126 ± 4 *	111 ± 4	<130 mg/dL
HDL-cholesterol (mg/dL)	68 ± 5	53 ± 3 *	44 ± 2 ***^,†^	>40/> 50 mg/dL
Leptin (ng/mL)	8.2 ± 1.8	61.6 ± 6.3 ***	43.5 ± 6.2 **	2.0–10.4 mg/dL
Uric acid (mg/dL)	4.3 ± 0.1	5.5 ± 0.3	6.1 ± 0.2 **	2.4–5.7 mg/dL
C-reactive protein (mg/L)	1.5 ± 0.5	9.7 ± 2.3 **	8.8 ± 1.4 *	<5 mg/L
Fibrinogen (mg/dL)	167 ± 34	385 ± 25 **	423 ± 18 ***	150–400 mg/dL
von Willebrand factor (%)	38 ± 2	157 ± 39 *	154 ± 10 *	75–125%
Homocysteine (µmol/L)	5.2 ± 0.4	9.1 ± 0.7	11.6 ± 1.1 *	0–12 μmol/L
Leucocytes (×10^9^)	6.7 ± 0.5	7.5 ± 0.5	7.8 ± 0.4	4.8–10.8 × 10^9^
Lymphocytes (%)	29 ± 2	30 ± 1	29 ± 1	20–50%
Neutrophils (%)	57 ± 2	61 ± 2	61 ± 1	45–75%
Monocytes (%)	8.7 ± 0.9	6.6 ± 0.3 *	7.1 ± 0.3 *	2–9%
Eosinophils (%)	4.5 ± 1.4	1.9 ± 0.4 **	2.6 ± 0.2 *	0–6 %
Basophils (%)	0.7 ± 0.1	0.4 ± 0.1	0.6 ± 0.1	0–%
Aspartate aminotransferase (U/L)	14 ± 1	22 ± 3	21 ± 2	<32 U/L
Alanine aminotransferase (U/L)	10 ± 4	29 ± 6 *	31 ± 3 *	<33 U/L
γ- glutamyltransferase (U/L)	12 ± 2	28 ± 6	44 ± 9	<40 U/L
Netrin-1 (pg/mL)	578 ± 33	924 ± 64 ***	841 ± 50 ***	-
Neogenin-1 (pg/mL)	2163 ± 180	1967 ± 143	1492 ± 129	-

DBP, diastolic blood pressure HOMA, homeostatic model assessment [fasting glucose (mmol/L) × fasting insulin (µU/mL)/22.5]; ND, not determined; OGTT, oral glucose tolerance test; QUICKI, quantitative insulin sensitivity check index [1/log fasting insulin (µU/mL) + log fasting glucose (mg/dl)]; SBP, systolic blood pressure. One-way ANOVA followed by Tukey’s *post hoc* tests or unpaired two-tailed Student’s *t* tests were used to analyze differences between groups where appropriate. * *p* < 0.05, ** *p* < 0.01 and *** *p* < 0.001 vs. lean. ^†^ *p* < 0.05, ^††^ *p* < 0.01 and ^†††^ *p* < 0.01 vs. obesity with normoglycaemia.

**Table 2:** Association between circulating concentrations of NTN-1 with anthropometric and biochemical characteristics.

	Circulating NTN-1 Levels	
	r	*p*
Age	0.04	0.738
Weight	**0.35**	**0.002**
Body mass index	**0.39**	**0.001**
Body fat	**0.34**	**0.004**
Waist circumference	**0.31**	**0.009**
Fasting glucose	−0.01	0.919
Fasting insulin	**0.40**	**0.001**
HOMA	**0.27**	**0.032**
QUICKI	−**0.26**	**0.037**
Triglycerides	0.12	0.350
Cholesterol	0.03	0.790
LDL-cholesterol	0.08	0.490
HDL-cholesterol	−0.24	0.059
Leptin	**0.36**	**0.035**
Fibrinogen	**0.29**	**0.046**
von Willebrand factor	0.07	0.719
C-reactive protein	0.20	0.251

HOMA, homeostatic model assessment; QUICKI, quantitative insulin sensitivity check index. Values are Pearson’s correlation coefficients (*r*) and associated *p* values. *p* values lower than 0.05 are highlighted in bold.

**Table 3:** Effects of weight loss in patients with OB after conventional diet and Roux-en-Y gastric bypass.

	Conventional Diet		RYGB	
	Before WL	After WL	Before WL	After WL
n (male, female)	14 (6, 8)	14 (6, 8)	23 (8, 15)	23 (8, 15)
Age (years)	47 ± 4	48 ± 4	43 ± 3	44 ± 3
Weight (kg)	95.1 ± 5.8	82.2 ± 4.7 ***	119.7± 4.7	80.8 ± 3.2 ^†††^
BMI (kg/m^2^)	34.2 ± 1.5	29.6 ± 1.2 ***	44.5± 1.2	30.0 ± 0.8 ^†††^
Body fat (%)	43.8 ± 1.2	37.1 ± 2.5 ***	52.1 ± 1.2	35.8 ± 1.7 ^†††^
Waist circumference (cm)	109.1 ± 3.8	97.6 ± 3.7 ***	127.3 ± 2.7	96.13 ± 2.4 ^†††^
Waist-to-hip ratio	0.97 ± 0.02	0.94 ± 0.03 *	0.96 ± 0.02	0.90 ± 0.02 ^††^
Fasting glucose (mg/dL)	94.2 ± 2.9	91.8 ± 1.9	110.4 ± 9.1	93.7 ± 5.6 ^††^
Fasting insulin (μU/mL)	10.9 ± 2.1	9.7 ± 2.1	18.4 ± 3.0	5.39 ± 1.4 ^††^
HOMA	2.61 ± 1.63	2.28 ± 1.60	5.64 ± 4.32	1.38 ± 1.58
QUICKI	0.344 ± 0.039	0.352 ± 0.050	0.315 ± 0.034	0.393 ± 0.052
Triglycerides (mg/dL)	120 ± 15	81 ± 8 **	131 ± 28	119 ± 50
Cholesterol (mg/dL)	202 ± 11	177 ± 7 *	200 ± 13	174 ± 15
LDL-cholesterol (mg/dL)	123 ± 10	108 ± 6	120 ± 11	90 ± 1
HDL-cholesterol (mg/dL)	54 ± 3	52 ± 3	50 ± 4	61 ± 5
Leptin (ng/mL)	26.86 ± 7	15.9 ± 3.9	31.5 ± 3.2	9.1 ± 1.2 ^†††^
Fibrinogen (mg/dL)	ND	ND	371 ± 20	350 ± 25
Homocysteine (µmol/L)	ND	ND	7.28 ± 0.43	6.12 ± 0.70
CRP (mg/L)	ND	ND	0.52 ± 0.1	0.08 ± 0.02 ^†^

BMI, body mass index; HOMA, homeostatic model assessment; QUICKI, quantitative insulin sensitivity check index; CRP, C-reactive protein; RYGB, Roux-en-Y gastric bypass; WL, weight loss. Data are mean ± SEM. ND, not determined. Differences between groups were analyzed by paired two-tailed Student’s *t* tests. * *p* < 0.05, ** *p* < 0.01, and *** *p* < 0.001 vs. before WL achieved by conventional diet; ^†^ *p* < 0.05, ^††^ *p* < 0.01, and ^†††^ *p* < 0.001 vs. before WL achieved by RYGB.

**Table 4:** Gene expression levels of inflammation- and extracellular matrix-related factors in human visceral adipose tissue.

Gene	Lean	Obesity with Normoglycaemia	Obesity with Type 2 Diabetes
*n*	7	31	24
*ADIPOQ*	1.00 ± 0.28	0.28 ± 0.05 **	0.33 ± 0.04 **
*ASC*	1.00 ± 0.19	1.44 ± 0.10 **	1.71 ± 0.11 ***
*IL1A*	1.00 ± 0.34	0.85 ± 0.12	1.43 ± 0.34
*IL1B*	1.00 ± 0.53	2.13 ± 0.33 *	6.25 ± 1.61 **^,^^†^
*IL6*	1.00 ± 0.40	5.08 ± 2.34 *	6.18 ± 1.06 ***^,^^†^
*MMP2*	1.00 ± 0.15	1.35 ± 0.16	1.68 ± 0.22
*MMP9*	1.00 ± 0.55	2.56 ± 0.74 *	3.76 ± 0.73 ***
*NLRP3*	1.00 ± 0.22	4.41 ± 0.77 ***	4.25 ± 0.54 ***
*NOD2*	1.00 ± 0.34	2.13 ± 0.61 *	2.52 ± 0.36 **
*TGFB*	1.00 ± 0.19	1.41 ± 0.13	2.36 ± 0.25 ***^,^^††^
*TNC*	1.00 ± 0.20	7.76 ± 1.77 **	6.91 ± 1.27 ***
*TNF*	1.00 ± 0.39	1.30 ± 0.15 *	1.40 ± 0.25

*ADIPOQ*, adiponectin; *ASC*, apoptosis-associated speck-like protein containing a CARD; *IL*, inte leukin; *MMP*, matrix metalloproteinase; *NLRP3*, NLR family pyrin domain containing 3; *NOD2*, nucleotide binding oligomerization domain containing protein 2; *TGFB*, transforming growth factor-β; *TNC*, tenascin C; *TNF*, tumor necrosis factor-α. Data represents the mean ± SEM of the ratio between the gene and *18S* rRNA expression levels. Differences between groups were analyzed by one-way ANOVA followed by Tukey’s *post hoc* test. * *p* < 0.05, ** *p* < 0.01 and *** *p* < 0.001 vs. lean. ^†^ *p* < 0.05 and ^††^ *p* < 0.01 vs. obesity with normoglycaemia.

## Data Availability

The data that support the findings of this study are available from the corresponding author upon reasonable request.

## Supplementary Materials

The following supporting information can be downloaded at: https://www.mdpi.com/article/10.3390/nu14204372/s1, Table S1: Sequences of the primers and TaqMan^®^ probes. Figure S1: Scatter diagrams showing the correlations of visceral adipose tissue (VAT) mRNA expression of netrin-1 (*NTN1*) and neogenin-1 (*NEO1*) between them and with body fat percentage (BF%). Figure S2: Effects of pro- and anti-inflammatory factors on neogenin-1 (*NEO1*) gene expression levels in human differentiated visceral adipocytes and monocyte-derived macrophages.
