# Supplementary material for: Netrin-1 Promotes Visceral Adipose Tissue Inflammation in Obesity and Is Associated with Insulin Resistance
Source: Nutrients. 2022 Oct 18;14(20):4372. doi: 10.3390/nu14204372 (PMC9611559; doi:10.3390/nu14204372)
Supplement: Supplementary file 1 [file nutrients-14-04372-s001.zip › nutrients-1964492-supplementary.pdf]

Table S1. Sequences of the primers and TaqMan® probes.

Gene (GenBank accession)	Oligonucleotide sequence (5’-3’)
ADIPOQ (NM_001177800)	
Forward	GGAGATCCAGGTCTTATTGGTCCTA
Reverse	CCTTGGATTCCCGGAAAGC
TaqMan® Probe	FAM-ACATCGGTGAAACCGGAGTACCCGG-TAMRA
ASC (NM_013258.4)	
Forward	AGCCAGGCCTGCACTTTATAGA
Reverse	CAGCAGCCACTCAACGTTTG
TaqMan® Probe	FAM-CACCGGGCTGCGCTTATCGC-TAMRA
CCL2 (NM_002982)	
Forward	GCTCATAGCAGCCACCTTCATT
Reverse	TCTGCACTGAGATCTTCCTATTGGT
TaqMan® Probe	FAM-TCGCTCAGCCAGATGCAATCAATGC-TAMRA
DCC (NM_005215.4)	
Forward	ACCCTAAATGAGCCGCCAAT
Reverse	GACACCAACGGTGACCACAAT
TaqMan® Probe	FAM-CACTCCTCAGAAGAACAGCAACCTGCTTGT -TAMRA
IL1A (NM_000575)	
Forward	GTTCTGAAGAAGAGACGGTTGAGTTT
Reverse	AAGTTGTATTTCACATTGCTCAGGAA
TaqMan® Probe	FAM-CATCGCCAATGACTCAGAGGAAGAAATCA-TAMRA
IL1B (NM_000576)	
Forward	CAGTGGCAATGAGGATGACTTG
Reverse	GTAGTGGTGGTCGGAGATTCGTA
TaqMan® Probe	FAM-TGGCCCTAAACAGATGAAGTGCTCCTTCC-TAMRA
IL6 (NM_000600)	
Forward	GCCCTGAGAAAGGAGACATGTAAC
Reverse	ATCCATCTTTTTCAGCCATCTTTG
TaqMan® Probe	FAM-AGGCACTGGCAGAAAACAACCTGAACC-TAMRA
IL32 (NM_001012631)	
Forward	GAGACAGTGGCGGCTTATTATGA
Reverse	GGCACCGTAATCCATCTCTTTCT
TaqMan® Probe	FAM-CAGCACCCAGAGCTCACTCCTCTACTTGAA-TAMRA
IL36 (NM_004530)	
Forward	TGTGGGACTTCCACGAAGTG
Reverse	CTTGCTCAAGAGCCTCTGGATAC
TaqMan® Probe	FAM-ACCCCAGTCACTGTTGCTGTTATCACATGC-TAMRA
MMP2 (NM_004530)	
Forward	CCATTTTGATGACGATGAGCTATG
Reverse	GTTGTACTCCTTGCCATTGAACAA
TaqMan® Probe	FAM-CTTGGGAGAAGGCCAAGTGGTCCGT-TAMRA
MMP9 (NM_004994)	
Forward	GCCCGGACCAAGGATACAGT
Reverse	CCCCTCAGTGAAGCGGTACA
TaqMan® Probe	FAM-ACGCGCTGGGCTTAGATCATTCCTCA-TAMRA
*NEO1 (*NM_001172623.1)	
Forward	GTGGAGCCCAACTGATAATCCTT

Reverse	CAATTTGATGAAGCGGGTAGAGA
TaqMan® Probe	FAM-AACATGCACCAGCCACAACGGGA-TAMRA
NLRP3 (NM_001079821.3)	
Forward	GTTCTGAAGAAGAGACGGTTGAGTTT
Reverse	AAGTTGTATTTCACATTGCTCAGGAA
TaqMan® Probe	FAM-CATCGCCAATGACTCAGAGGAAGAAATCA-TAMRA
NOD2 (NM_001293557)	
Forward	TTCAGGAATTACCAGTCCCATTG
Reverse	GGTCCTCAGCTTGGCCATATACT
TaqMan® Probe	FAM-CCCTGCCTTTGGAAGCTGCCACA-TAMRA
NTN1 (NM_000575)	
Forward	AGGGCTACTACCGCGACATG
Reverse	ACACTGGCCGGTGGTTTG
TaqMan® Probe	FAM-TGCAAAGCCTGTGATTGCCACCC-TAMRA
SEMA3E (NM_001178129.2)	
Forward	CGAGGTGGAATGGCTCAAGA
Reverse	TCGATGGTGAGCAGGAAGTTG
TaqMan® Probe	FAM-AGGATGTCATCGACCCCACCCAGG-TAMRA
SPP1 (NM_000582)	
Forward	CATCCAGTACCCTGATGCTACAGA
Reverse	GGCCTTGTATGCACCATTCAA
TaqMan® Probe	FAM-ACATCACCTCACACATGGAAAGCGAGGA-TAMRA
TGFB (NM_000660)	
Forward	GCCCAGCATCTGCAAAGC
Reverse	TCCTTGCGGAAGTCAATGTACA
TaqMan® Probe	FAM-CACCAACTATTGCTTCAGCTCCACGGA-TAMRA
TNC (NM_002160.4)	
Forward	GCCCAGCATCTGCAAAGC
Reverse	TCCTTGCGGAAGTCAATGTACA
TaqMan® Probe	FAM-CACCAACTATTGCTTCAGCTCCACGGA-TAMRA
TNF (NM_000594)	
Forward	CCCCAGGGACCTCTCTCTAATC
Reverse	ACATGGGCTACAGGCTTGTCA
TaqMan® Probe	FAM-CCTCTGGCCCAGGCAGTCAGATCAT-TAMRA
UNC5B (NM_001244889.2)	
Forward	AGAGTCGCCGAGCCTACGT
Reverse	TTGCCCAGAGGCTCCTGAT
TaqMan® Probe	FAM-CGCATCGCCTACCTGCGCAAG-TAMRA

ADIPOQ, adiponectin; *ASC,* apoptosis-associated speck-like protein containing a CARD*; CCL2*, monocyte chemoattractant protein-1; *DCC*, deleted in colorectal cancer; *IL*, interleukin; *MMP*, matrix metalloproteinase; *NEO1*, neogenin-1; *NLRP3*, NLR family pyrin domain containing 3; *NOD2*, nucleotide-binding oligomerization domain containing protein 2; *NTN1*, netrin-1; *SEMA3E*, semaphoring 3E; *SPP1*, osteopontin; *TGFB*, transforming growth factor-β; *TNC,* tenascin C*; TNF*, tumour necrosis factor-α; *UNC5B,* UNC-5 netrin receptor B.

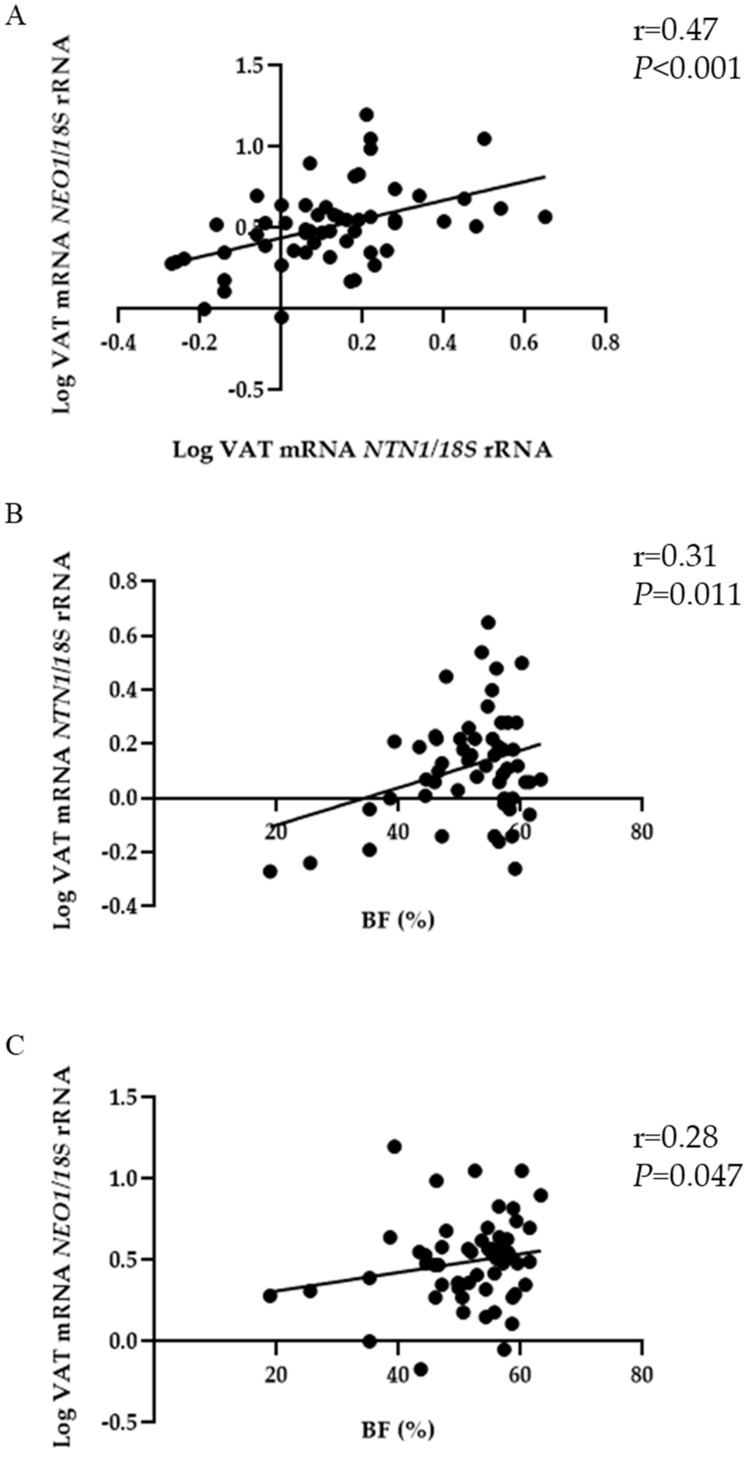

Figure S1. Scatter diagrams showing the correlations of visceral adipose tissue (VAT) mRNA expression of netrin-1 (*NTN1*) and neogenin-1 (*NEO1*) between them (A) and with body fat percentage (BF%) (B, C). Pearson's correlation coefficient (r) and *P* values are indicated.

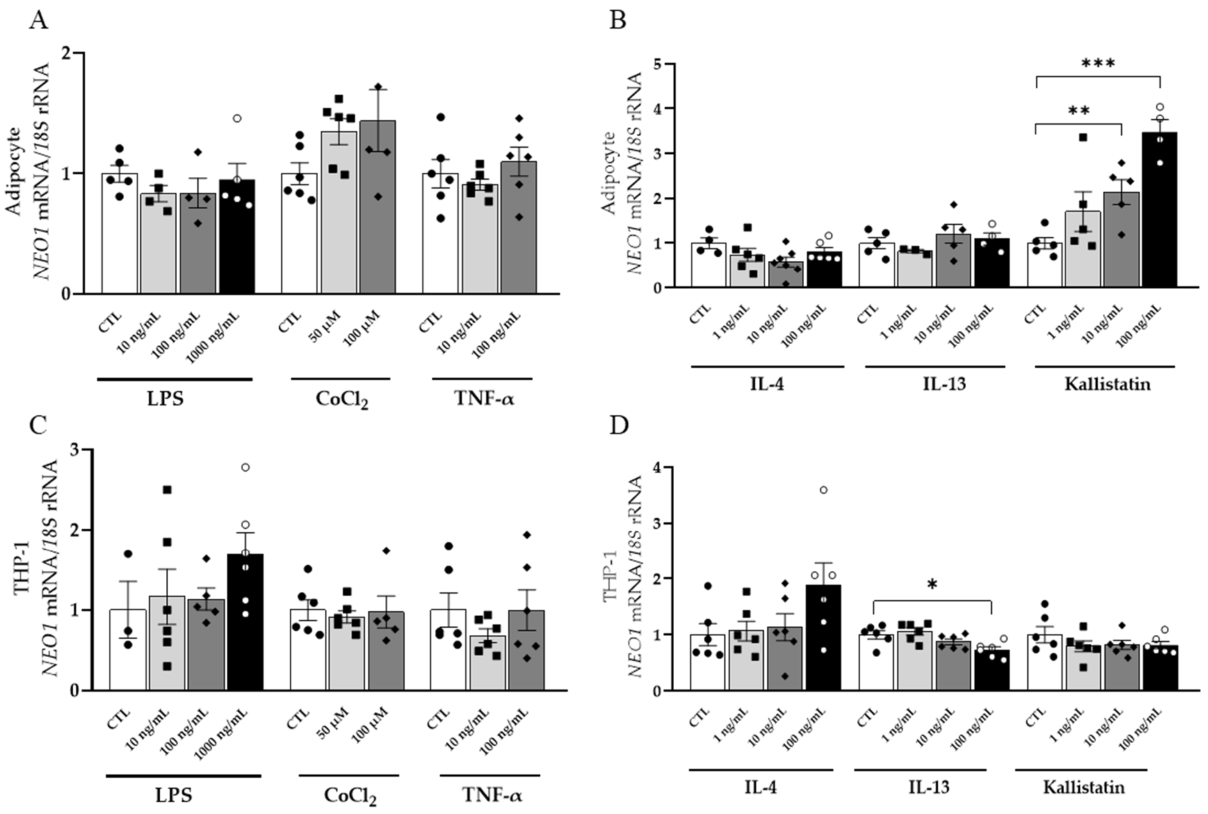

Figure S2. Effects of the pro-inflammatory factors lipopolysaccharide (LPS), cobalt chloride ($CoCl_2$) and tumour necrosis factor (TNF)-α (A, C) and the anti-inflammatory mediators interleukin (IL)-4, IL-13 and kallistatin (B, D) on neogenin-1 (*NEO1*) gene expression levels in human differentiated visceral adipocytes and monocyte-derived macrophages. Gene expression levels in unstimulated cells were assumed to be 1. Differences between groups were analysed by one-way ANOVA followed by Dunnett's *post hoc* tests. $^{*}P<0.05$, $^{**}P<0.01$ and $^{***}P<0.001$.
